# Recursive Settling of Particles in Shear Thinning Polymer Solutions: Two Velocity Mathematical Model

**DOI:** 10.3390/polym14194241

**Published:** 2022-10-10

**Authors:** Vladimir Neverov, Vladimir Shelukhin

**Affiliations:** Lavrentyev Institute of Hydrodynamics, 630090 Novosibirsk, Russia

**Keywords:** suspensions, two-velocity continua, shear thinning fluid, recursive sedimentation, tilted vessel

## Abstract

Processing of the available experimental data on particles settling in shear-thinning polymer solutions is performed. Conclusions imply that sedimentation should be recursive, since settling also occurs within the sediment. To capture such an effect, a mathematical model of two continua has been developed, which corresponds to experimental data. The model is consistent with basic thermodynamics laws. The rheological component of this model is a correlation formula for gravitational mobility. This closure is justified by comparison with known experimental data available for particles settling in vertical vessels. In addition, the closure is validated by comparison with analytical solutions to the Kynch one-dimensional equation, which governs dynamics of particle concentration. An explanation is given for the Boycott effect and it is proven that sedimentation is enhanced in a 2D inclined vessel. In tilted vessels, the flow is essentially two-dimensional and the one-dimensional Kynch theory is not applicable; vortices play an important role in sedimentation.

## 1. Introduction

Particulate fluids are common in both natural and industrial processes. Fiber-reinforced polymers, detergents, blood, and drilling muds are some examples where particles are present in a suspending fluid [1,2]. Sedimentation of suspensions in complex fluids is of interest in technological operations, such as oil and gas exploration. Many drilling muds are polymer-based fluids with shear-thinning rheology manifesting itself through a loss of apparent viscosity with increasing strain-rates. Operational stops occur frequently during oil-drilling processes. Interruption of drilling mud pumping causes the particles to settle through the annular space. This may give rise some undesirable phenomena in drilling operations, such as a stuck pipe. The understanding of suspension dynamics in complex fluids is also important for applications in particle manipulation in microfluidic devices [3]. The methods and numerical algorithms developed in the present paper contribute to enhancing knowledge of the processes and their optimization in the fields of oil exploration and microfluidic devices.

Laboratory study based on the gamma-ray attenuation technique reveals significant differences in the behavior of particles settling in Newtonian and non-Newtonian fluids [4]. Particularly, control of the concentration of solids proves that sediment is formed faster in shear-thinning fluid when compared to a Newtonian fluid of similar apparent viscosity.

In order to develop a theory of settling, it is first necessary to understand the interaction of a single particle with a carrier fluid and the mutual interaction between two particles.

Clearly, the dynamics of a particles can be strongly affected by the rheology of the interstitial fluid. In Ref. [5], some problems involving rigid spherical particles in shear-thinning fluids are considered in the absence of inertia; for settling particles, analytical formulas are derived and differences in comparison to a Newtonian fluid are demonstrated. Experiments on sedimentation in non-Newtonian shear thinning fluids were considered in Ref. [6] in order to validate a certain empirical dependence of the particle settling velocity on the volume concentration of particles. It should be noted that, based on one-velocity continuum models, the existing tools of computational fluid dynamics make it possible to solve sedimentation problems, taking into account many complex processes. It is worth mentioning the works [7,8,9], where the sedimentation of particles in stirred vessels is analyzed in the case of two-phase turbulent mixing flows, taking into account chemical reactions, crystallization, and dissolution processes.

In this work, a different approach is used. In order to take into account particle–fluid and particle–particle interactions, particles are considered as a phase, which can be described within the continuum mechanics approach. As a result, the entire suspension becomes a two-velocity continuum, with the fluid and the solid phases enjoying some rheological constitutive laws. Such a method was validated in the recent paper concerning the Boycott sedimentation effect, which stated that enhanced sedimentation occurs in a tilted vessel [10].

In a great number of papers [11], particles and the carrier fluid are also assumed to be two different phases. Let us highlight the differences and similarities between our model and other two-phase approaches. First of all, the equations used are consistent with the laws of thermodynamics. The article by A.S. Baumgarten and K. Kamrin [12] also refers to the two-phase model, which is consistent with thermodynamics, but the method and equations are different. To achieve compliance with the laws of thermodynamics in the case of concentrated suspensions, the ideas that were proposed for the mathematical description of the superfluidity of liquid helium II in the papers of L. D. Landau and I. M. Khalatnikov [13,14] are applied.

An important point of the theory is that interaction forces between phases for reversible processes without dissipation effects can be uniquely identified while reconciling the energy conservation equation, both with other conservation laws and the basic thermodynamic principles. As far as dissipative processes are concerned, the interaction forces are introduced, fitting the general deGroot–Mazur principles of irreversible thermodynamics [15]. The Landau–Khalatnikov thermodynamic method finds applications in the description of multi-phase flows [16], particularly in studying fluid-saturated poroelastic media, both with the use of a two-velocity [17] and a three-velocity continuum [18]. Recently, it was established in Ref. [19] that the same approach can be applied in building up conservation laws for suspensions with rotating particles. In the present paper, we extend the Landau–Khalatnikov approach by formulating the diffusion equation for the mass concentration of particles involving a generalized Fick’s law for the concentration flux vector in such a way that it depends not only on the concentration gradient and gravitation vector scaled by a mobility factor, but on the pressure gradient, temperature gradient, and gradient of modulus of the slip velocity as well.

The principle goal of the present paper is to adjust the method of [10] for the description of particles settling in a polymer solution. To this end, particles are assumed to be suspended in a non-Newtonian power-law fluid, in contrast to [10], where such a fluid is considered to be Newtonian. One more rheological feature of this work is that the gravitational mobility in Fick’s law is chosen in such a way that the numerical results on sedimentation are consistent with experiments on particles settling in polymer solutions [4]. In fact, a suitable correlation is proposed for the dependence of mobility on particle concentration. The proposed arguments are based on the Kynch theory, in which the sedimentation of a dense suspension is considered as a concentration wave [20]. A zone with a high concentration of particles is first formed at the bottom of a vessel; then, this zone propagates upward like a shock wave. Some successful applications of this theory can be found in Refs. [21,22]. The calculations performed in this work show that the sedimentation of particles in polymer solutions is recursive, since settling is also observed inside the sediment. Sedimentation in a tilted vessel is considered and it is proven that the Boycott effect also holds in the polymer solution. A comparison with Newtonian carrier fluid is performed.

## 2. Mathematical Model

Let us consider a joint flow of two continua when an arbitrary volume *V* contains a fluid (index *f*) and a granular phase (index *s*). Volume, mass, and pressure of the fluid and the granular phases are denoted by $V_{f},m_{f},p_{f}$, and $V_{s},m_{s},p_{s}$, respectively. It is assumed that the granular phase is a mixture of dry particles and a carrier fluid such as proppant and gel, for example. In this case, $V_{s}=V_{M}+V_{p}$ and $m_{s}=m_{M}+m_{p}$. The particles are “frozen” in the carrier liquid; i.e., the granular phase is characterized by just one speed $v_{s}$, one viscosity, and one stress tensor. In what follows, the indexes *f* and *s* stand for fluid and solid phases, respectively.

The quantities

(1) $$\rho =\frac{m}{V},\;\rho_{s}=\frac{m_{s}}{V},\;\rho_{f}=\frac{m_{f}}{V},\;\rho_{p}=\frac{m_{p}}{V},\;\phi_{j}=\frac{V_{j}}{V},\;\rho_{M}=\frac{m_{M}}{V},\;c=\frac{m_{p}}{m}.$$

are assigned to the unit volume. Here, $c=\rho_{p}/\rho$ is the particle mass concentration and $\phi_{j}$ is the volume fraction of the *j*-phase with j=f,p,M. It follows from the above definitions that the partial densities $\rho_{j}$ are related to the material densities ${\bar{\rho }}_{j}$ by the following formulas

$$\rho_{j}=\phi_{j}{\bar{\rho }}_{j},\;{\bar{\rho }}_{j}\equiv \frac{m_{j}}{V_{j}},\;\phi_{f}+\phi_{s}=1,\;\phi_{s}=\phi_{p}+\phi_{M},\;\rho =\rho_{f}+\rho_{p}+\rho_{M}.$$

Generally, the phase pressures $p_{s}$ and $p_{f}$ are different. However, as in Ref. [19], it is assumed that $p_{s}=p_{f}=p$. Such a hypothesis works well when the surface tension at the boundaries separating the phases is negligible.

Let $v_{i}$, $T_{i}$, l, *k* stand for the velocity, the viscous part of the stress tensor, the particle concentration flux vector, and the interface friction coefficient, respectively.

In what follows, we use the tensor notations. Given two vectors a and b, we define the scalar product a·b= $a_{i}b_{i}$. The tensor product a⊗b is a matrix such that ${\left(a⊗b\right)}_{ij}=$ $a_{i}b_{j}$. The matrix $A^{*}$ stands for the adjoint matrix of *A*, i.e., ${\left(A^{*}\right)}_{ij}={\left(A\right)}_{ji}$. The *i*-th component of the vector divA is defined by the formula ${\left(\mathrm{div}\;A\right)}_{i}=\partial A_{ik}/\partial x_{k}$.

Neglecting the rotation of particles and thermal effects, it follows from [19] that the mathematical model

(2) $$\frac{\partial (\rho_{s}v_{s})}{\partial t}+\mathrm{div}\;\left(\rho_{s}v_{s}⊗v_{s}\right)=-\frac{\rho_{s}}{\rho }\nabla p-\frac{\rho_{s}\rho_{f}}{2\rho }\nabla u^{2}-k\;u+\mathrm{div}\;T_{s}+\rho_{s}g\;,$$

(3) $$\frac{\partial (\rho_{f}v_{f})}{\partial t}+\mathrm{div}\;\left(\rho_{f}v_{f}⊗v_{f}\right)=-\frac{\rho_{f}}{\rho }\nabla p+\frac{\rho_{s}\rho_{f}}{2\rho }\nabla u^{2}+k\;u+\mathrm{div}\;T_{f}+\rho_{f}g,$$

(4) $$\frac{\partial (\rho c)}{\partial t}+\mathrm{div}\;\left(cj+l\right)=0,$$

(5) $$\rho_{st}+\mathrm{div}\;\left(\rho_{s}v_{s}\right)=0,\;\rho_{ft}+\mathrm{div}\;\left(\rho_{f}v_{f}\right)=0,\;$$

for six unknown functions $\rho_{s}$, $\rho_{f}$, *p*, *c*, $v_{s}$, $v_{f}$ can be derived. Here, p=p(ρ) is the prescribed state equation, g is the gravitation vector, and

$${\left(\nabla \;p\right)}_{i}=\frac{\partial p}{\partial x_{i}},\;j=\rho_{s}v_{s}+\rho_{f}v_{f},\;\rho =\rho_{s}+\rho_{f},u=v_{s}-v_{f},\;\mathrm{div}\;l\equiv \frac{\partial l_{i}}{\partial x_{i}},\;u^{2}=u\cdot u.$$

Rheological assumptions are formulated as follows. Given a velocity field v(x), the corresponding rate of strain tensor *D* is defined by the formula

$$D=\frac{\nabla v+{\left(\nabla v\right)}^{*}}{2},\;{\left(\nabla v\right)}_{ij}=\frac{\partial v_{i}}{\partial x_{j}}.$$

The fluid phase is considered to be a non-Newtonian fluid. This implies that

(6) $$T_{f}=2\eta_{f}D_{f},$$

with the power-law viscosity

(7) $$\eta_{f}=\eta_{f}^{0}{\dot{\gamma }}^{n-1},$$

where the power *n* satisfies the shear thinning condition 0<n<1. Here, $\eta_{f}^{0}$ is the consistency and $\dot{\gamma }$ is the dimensionless shear strain:

(8) $$\dot{\gamma }=\frac{\sqrt{2D_{f}:D_{f}}}{\omega },\;D:D=D_{ij}D_{ij},$$

with ω being the reference frequency. The shear stress $\tau =\sqrt{T_{f}:T_{f}/2}$ satisfies the equality

(9) $$\tau =\omega \eta_{f}^{0}{\dot{\gamma }}^{n}.$$

Given the volume fraction of the solid phase $\phi_{s}$, the rheology of the solid phase is defined by the Newtonian law

(10) $$T_{s}\left(\phi_{s}\right)=2\eta_{s}\left(\phi_{s}\right)D_{s}.$$

Here,

(11) $$\eta_{s}\left(\phi_{s}\right)=\eta_{s}^{0}{\left(1-\frac{\phi_{s}}{\phi_{s}^{*}}\right)}^{-2.5}$$

is the viscosity given by the Krieger–Douhgerty empirical closure [23], with $\phi_{s}^{*}$ and $\eta_{s}^{0}$ being the maximal reference value of $\phi_{s}$ and the consistency, respectively.

One more rheological equation is the Fick law [10]:

(12) $$l=-\left(\gamma_{3}\nabla c+\gamma_{1}\nabla p+\gamma_{4}\nabla \;u^{2}\right)+\rho cBg.$$

Due to the mass conservation laws (5), Equations (2) and (3) reduce to

(13) $$\rho_{s}\left(\frac{\partial v_{s}}{\partial t}+v_{s}\cdot \nabla v_{s}\right)=-\frac{\rho_{s}}{\rho }\nabla p-\frac{\rho_{s}\rho_{f}}{2\rho }\nabla u^{2}-k\;u+\mathrm{div}\;T_{s}+\rho_{s}g\;,$$

(14) $$\rho_{f}\left(\frac{\partial v_{f}}{\partial t}+v_{f}\cdot \nabla v_{f}\right)=-\frac{\rho_{f}}{\rho }\nabla p+\frac{\rho_{s}\rho_{f}}{2\rho }\nabla u^{2}+k\;u+\mathrm{div}\;T_{f}+\rho_{f}g,$$

where ${\left(v\cdot \nabla v\right)}_{i}=v_{j}\partial v_{i}/\partial x_{j}.$ Such equations are of use in the numerical calculations performed below.

## 3. Incompressible Isothermal Flows

Let us formulate a hypothesis of incompressibility. It is assumed that the mud volume fraction $\phi_{M}$ is negligible and the densities of materials ${\bar{\rho }}_{f}$, ${\bar{\rho }}_{p}$ and ${\bar{\rho }}_{M}$ are constants. Then, it follows from (1) that

(15) $$\rho_{s}\approx c\rho ,\;\rho_{f}\approx \left(1-c\right)\rho ,\;\phi_{s}=\frac{c}{R_{0}+c\left(1-R_{0}\right)},\;\phi_{f}=\frac{R_{0}\left(1-c\right)}{R_{0}+c\left(1-R_{0}\right)},$$

where $R_{0}={\bar{\rho }}_{s}/{\bar{\rho }}_{f}$. Observe that the total density ρ and the partial densities $\rho_{j}$ are not constant in contrast to the densities of the materials. By the incompressibility assumption, one can easily derive the following formulas:

$$\frac{\rho_{s}}{{\bar{\rho }}_{f}}=c\left[1+\left(R_{0}-1\right)\phi_{s}\left(c\right)\right]\equiv r_{s}\left(c\right),\;\frac{\rho_{f}}{{\bar{\rho }}_{f}}=1-\phi_{s}\left(c\right)\equiv r_{f}\left(c\right),\;\rho =\frac{{\bar{\rho }}_{s}}{R_{0}+c\left(1-R_{0}\right)}.$$

The functions $r_{s}\left(c\right)$ and $r_{f}\left(c\right)$ are dimensionless partial densities.

One more consequence of the incompressibility assumption is that the volumetric mean velocity is divergence-free:

(16) $$\mathrm{div}\;v=0,\;v\equiv \phi_{s}\left(c\right)v_{s}+\phi_{f}\left(c\right)v_{f}.$$

Equation (4) is equivalent to

(17) $$\frac{\partial c}{\partial t}+\tilde{v}\cdot \nabla \;c+\rho^{-1}\left(c\right)\mathrm{div}\;l=0,\;\tilde{v}\equiv cv_{s}+\left(1-c\right)v_{f},$$

where $\tilde{v}$ is the mean mass velocity. Thus, a mathematical model for four unknown functions *p*, *c*, $v_{s}$ $v_{f}$ is derived that obeys the Equations (13)–(17). The parameters $\eta_{s}$, $\eta_{f}$, *k*, $\gamma_{j}$ are assumed to be known functions of the mass concentration *c*.

Under the incompressibility hypothesis, pressure is no longer a thermodynamic parameter and does not satisfy the equation of state. It is now included in the list of unknown functions, as in the case of Navier–Stokes models of a viscous incompressible fluid. Densities can be restored from equalities (15).

The diffusion coefficients $\gamma_{j}$ vanish when any phase disappears. As for the friction, we use the correlation formula

(18) $$k\left(c\right)=\frac{3}{4}C_{D}\frac{c{\bar{\rho }}_{f}\left|u\right|}{d_{p}},$$

proposed in Ref. [24], where $d_{p}$ is the particle diameter and $C_{D}$ is the particle/fluid friction:

$$C_{D}=\left\{\begin{array}{cc} \frac{24}{Re_{p}}\left(1+0.15Re_{p}^{0.678}\right) & \mathrm{if}\;Re_{p}<1000,\\ 0.44 & \mathrm{if}\;Re_{p}>1000, \end{array}\right.Re_{p}=\frac{d_{p}{\bar{\rho }}_{f}\left|u\right|}{\eta_{f}^{0}}.$$

For the case of sedimentation, the particle Reynolds number $Re_{p}$ is very small, $Re_{p}<<1$, and one can use the following approximation $C_{D}=24/Re_{p},\;k=18\eta_{f}^{0}c/\left(d_{p}^{2}\right).$

With *g* being the gravitation acceleration, the formula g= $-ge_{y}$ is valid where $e_{y}={\left(0,1\right)}^{T}$. The domain $\Omega_{0}$ denotes the vertical cell $\{0<x<a_{1},\;0<y<a_{2}\},\;$ with the *y*-axis directed upwards. In what follows, flows are considered in the tilted cell $\Omega_{\alpha }$, with the inclination angle α measured from $e_{y}$.

Given the reference values $a_{1}$, *V*, $l_{0}$, $t_{0}$, and $\bar{p}$, the dimensionless variables are defined as follows

(19) $$x^{\prime }=\frac{x}{a_{1}},\;y^{\prime }=\frac{y}{a_{1}},\;v^{\prime }=\frac{v}{V},\;p^{\prime }=\frac{p}{\bar{p}},\;l^{\prime }=\frac{l}{l_{0}},\;t^{\prime }=\frac{t}{t_{0}},$$

with the assumptions

$$t_{0}=\frac{a_{1}}{V},\;\omega =\frac{V}{a_{1}},\;l_{0}={\bar{\rho }}_{f}V,\;\bar{p}={\bar{\rho }}_{f}ga_{1}.$$

As a result of passage to dimensionless variables, the following dimensionless parameters and functions appear:

(20) Re=a1Vρ¯fηf0,k1=18ηf0a1ρ¯fVdp2,Fr=ga1V2,

(21) λ=Fr·Re=ρ¯fa12gηf0V,β=k1·Re=18a12dp2,

(22) $$\Gamma_{1}\left(c\right)=\frac{\gamma_{1}\left(c\right)\bar{p}}{l_{0}a_{1}},\;\Gamma_{3}=\frac{\gamma_{3}}{a_{1}l_{0}},\;\Gamma_{4}\left(c\right)=\frac{\gamma_{4}\left(c\right)V^{2}}{a_{1}l_{0}},\;\Gamma_{5}\left(c\right)=\frac{cr(c)B(c)g}{V}.$$

In the new variables, the rheological equations become $\dot{\gamma }=\sqrt{2D_{f}^{\prime }:D_{f}^{\prime }}$,

(23) $$T_{f}=\frac{\eta_{f}^{0}V}{a_{1}}T_{f}^{\prime },\;T_{f}^{\prime }=2{\dot{\gamma }}^{n-1}D_{f}^{\prime },\;T_{s}=\frac{\eta_{f}^{0}V}{a_{1}}T_{s}^{\prime },\;T_{s}^{\prime }=2\frac{\eta_{s}}{\eta_{s}^{0}}\frac{\eta_{s}^{0}}{\eta_{f}^{0}}D_{s}^{\prime }.$$

Omitting the primes, we find that the functions $v_{f}^{\prime }\left(x^{\prime },y^{\prime },t^{\prime }\right)$, $v_{s}^{\prime }\left(x^{\prime },y^{\prime },t^{\prime }\right)$, $c(x^{\prime },y^{\prime },t^{\prime })$, and $p(x^{\prime },y^{\prime },t^{\prime })$ satisfy the equations

(24) rsRedsvsdt=−rsλr∇p+ηs0ηf0div2ηsηs0Ds−βcu−rsrfRe2r∇u2−λrs·ey,

(25) rfRedfvfdt=−rfλr∇p+div2γ˙n−1Df+βcu+rfrsRe2r∇u2−λrfey,

(26) $$\mathrm{div}\;v=0,\;v\equiv \phi_{s}\left(c\right)v_{s}+\phi_{f}\left(c\right)v_{f}.$$

(27) $$\frac{dc}{dt}+\frac{\mathrm{div}\;l}{r}=0,\;l=-\left(\Gamma_{3}\nabla c+\Gamma_{1}\nabla p+\Gamma_{4}\nabla \;u^{2}\right)-\Gamma_{5}e_{y},$$

in the domain

(28) $$\Omega_{\alpha }=\left\{x=X\cos\alpha +Y\sin\alpha ,\;y=-X\sin\alpha +Y\cos\alpha \right\},$$

where 0<X<1, $0<Y<h=a_{2}/a_{1}$. Let us formulate boundary and initial conditions:

(29) $$\partial \Omega_{\alpha }:\;v_{s}=0,\;v_{f}=0,\;l\cdot n=0,\;\nabla p\cdot n=0,$$

(30) $$t=0:\;v_{s}=v_{s0},\;v_{f}=v_{f0},\;c=c_{0}.$$

The diffusion coefficients $\gamma_{1}\left(c\right)$ and $\gamma_{4}\left(c\right)$ vanish at c=0 and c=1. This is why it is reasonable to set $\Gamma_{i}=\Gamma_{i}^{0}c\left(1-c\right),$ where $\Gamma_{i}^{0}$ are constants, i=1,4.

## 4. Gravitation Mobility of Particles in a Solution of Polymers

First, we consider settling in a Newtonian fluid where n=1 and $\eta_{f}=\eta_{f}^{0}$. For simplicity, it is assumed that ρ=const and that the gravitation diffusion is dominant; i.e., $\gamma_{i}=0$∀i. In dimension variables, it follows from (4) and (12) that concentration obeys the equation

(31) $$\frac{\partial c}{\partial t}+\mathrm{div}\;\left[c\left(\tilde{v}-gB\left(c\right)e_{y}\right)\right]=0.$$

On the other hand, some authors [25] apply the equation

(32) $$\frac{\partial c}{\partial t}+\mathrm{div}\;\left(cv_{p}\right)=0$$

while addressing sedimentation. Here, $v_{p}=\tilde{v}+v_{slip}$ is the particle velocity and

(33) $$v_{slip}=-V_{St}H\left(c\right)e_{y},\;V_{St}=\frac{2\left({\bar{\rho }}_{p}-{\bar{\rho }}_{f}\right)g{\left(d_{p}/2\right)}^{2}}{9\eta_{f}^{0}},$$

where $V_{St}$ is the Stokes settling velocity in Newtonian fluid and H(c) is the hindered settling function [25]. Comparing (31) and (32), we find that

(34) $$B\left(c\right)=\frac{V_{St}H\left(c\right)}{g}=2\left({\bar{\rho }}_{p}-{\bar{\rho }}_{f}\right){\left(\frac{d_{p}}{2}\right)}^{2}\frac{H(c)}{9\eta_{f}^{0}}.$$

As shown in Section 6, the Richardson–Zaki closure

(35) $$H_{rz}\left(c\right)={\left(1-c\right)}^{m}$$

for the function H(c) meets the settling in a Newtonian fluid with *m* = 4.65 [26].

As for shear thinning non-Newtonian fluids, it is proven in Section 6 that the shear thinning closure

(36) $$H_{sh}\left(c\right)=a{\left(1-c\right)}^{k}+bc^{m-1}\left(1-c\right)-d{\left(1-c\right)}^{l}.$$

for the function H(c) with the special choice (46) of the constants a,b,d,k,m and *l* fits the experiment data in Ref. [4] better than any hindered settling function of the form (35). In what follows, the functions $B_{rz}\left(c\right)$ and $B_{sh}\left(c\right)$ are defined by Equation (34) with the hindered settling function H(c) given by Equations (35) or (36), respectively.

## 5. Settling in 2D Tube

Here, we apply the mobility closure Equation (34) with the hindered settling function (36) to particles settling in polymer solutions governed by the rheology of the shear thinning fluids. Particularly, we consider sedimentation in inclined vessels on the bases of the two-velocity model (24)–(27) and establish the Boycott effect.

**Figure 1:**
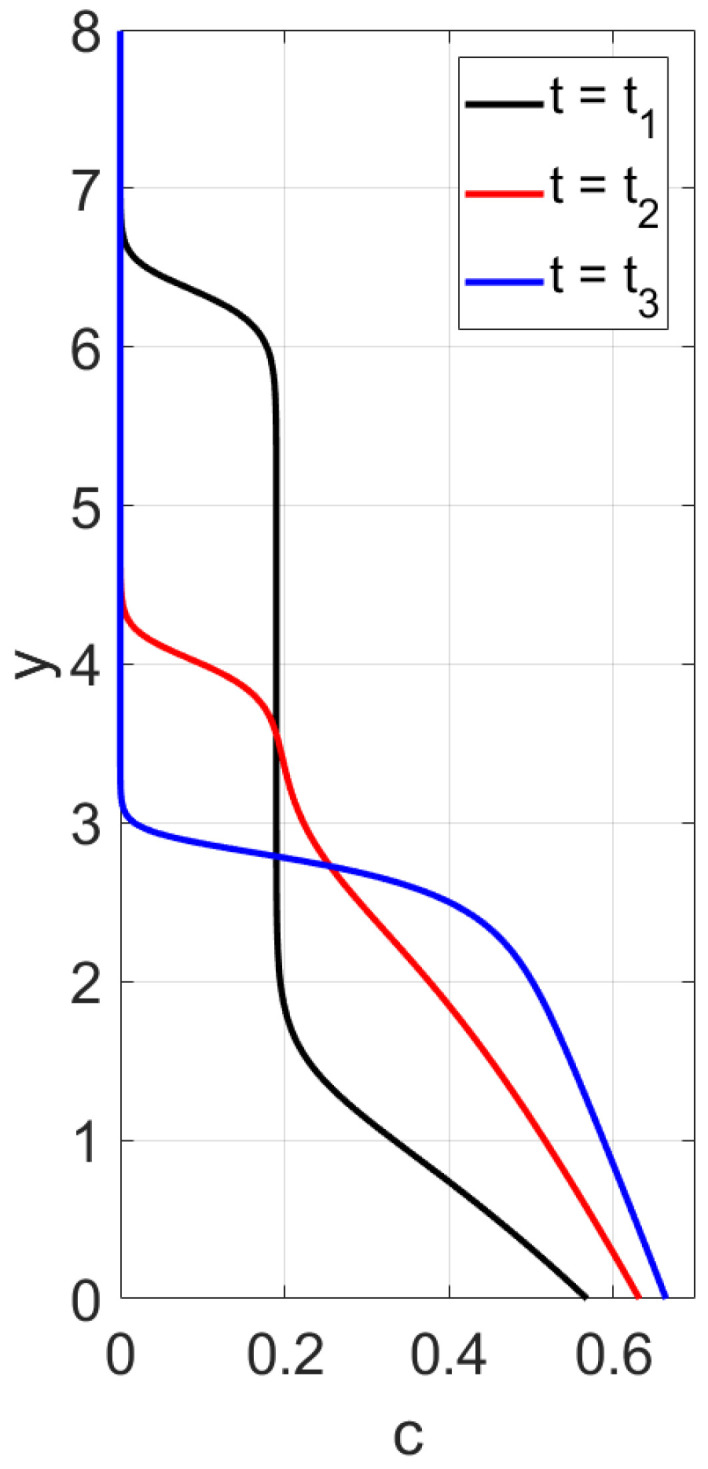
Calculated profiles of average concentration over the cross section of the 2D tube for different time instances in a vertical vessel in the case of mobility $B_{rz}$.

**Figure 2:**
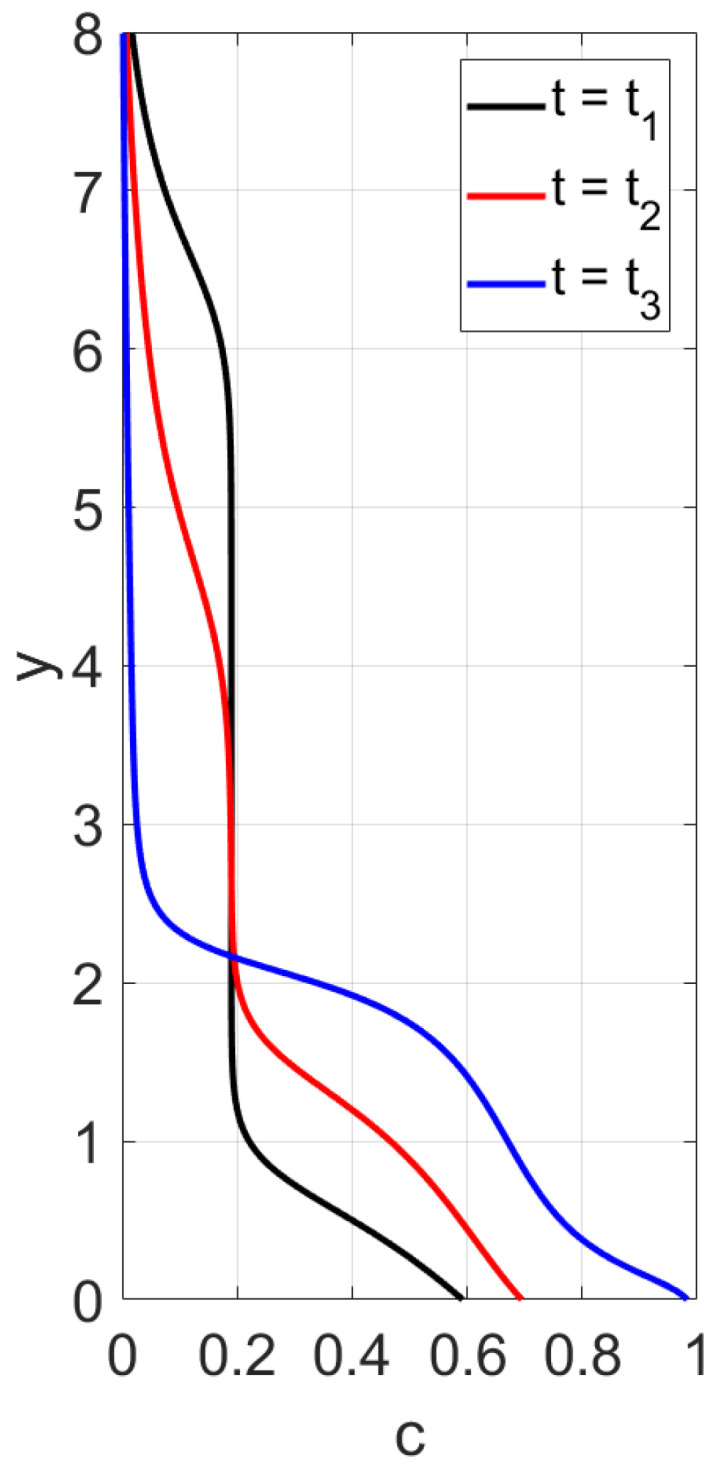
Calculated profiles of average concentration over the cross section of the 2D tube for different time instances in a vertical vessel in the case of the shear thinning mobility $B_{sh}$. Effect of two-fold particle sedimentation.

**Figure 3:**
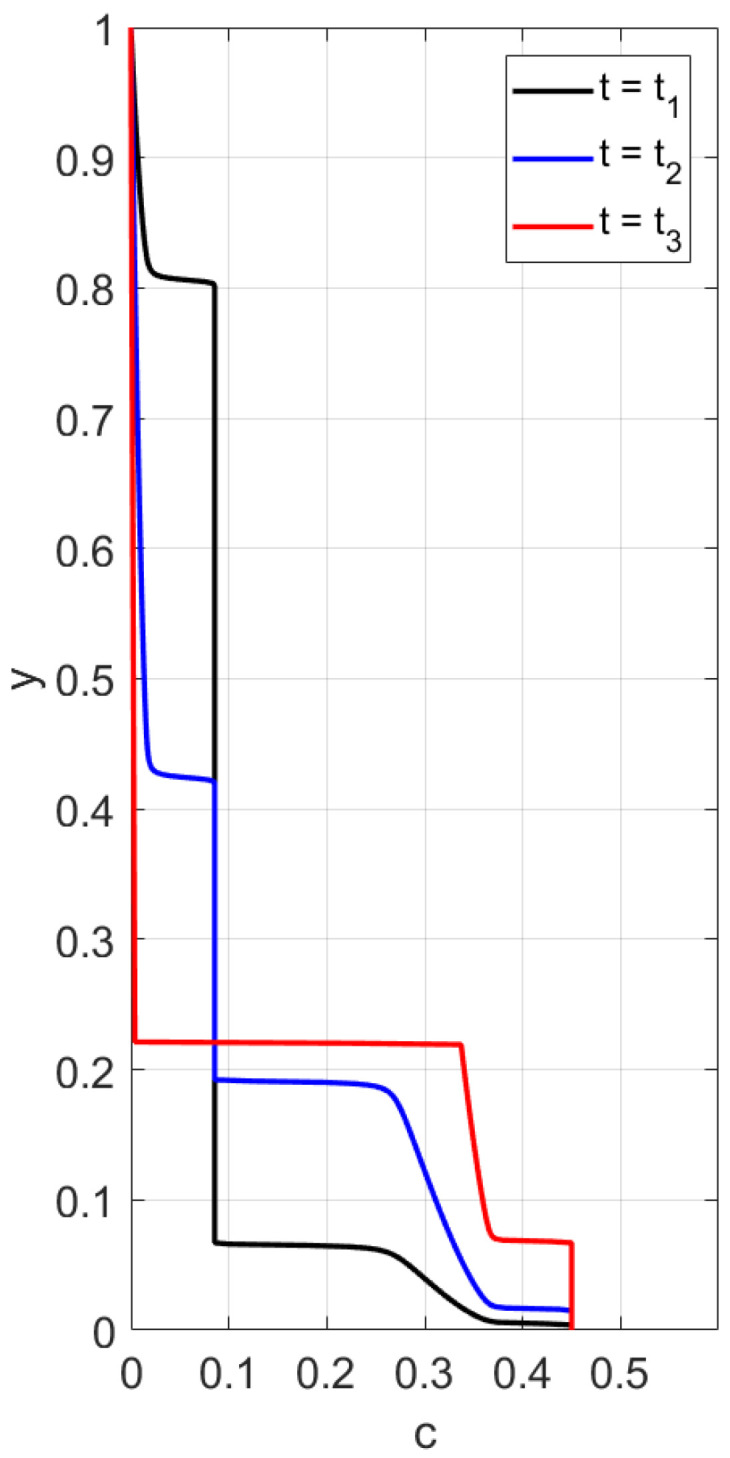
Calculated profiles of the concentration wave at different time instances for the shear thinning flux $F_{sh}\left(c\right)$ in the case when all the dissipation effects are negligible. The initial and boundary data are $c_{0}=0.09$, $c_{bot}=1$, $c_{top}=0$.

First, sedimentation in a vertical vessel is considered. Calculations reveal that the concentration structures are different for the mobilities $B_{rz}$ or $B_{sh}$. Figure 1 is obtained with the use of the mobility $B_{rz}$ and shows that there are two concentration waves smoothed by the diffusion effect, which propagate in opposite directions. The downward wave starts from the top of the vessel and leaves no particles behind, whereas the wave going up describes an increasing zone of sediment. As for the non-Newtonian fluid with the shear thinning mobility $B_{sh}$, there are three waves, as shown in Figure 2. At the initial stage, only two waves moving towards each other are observed. Then, a third wave appears, going from bottom to top. This wave indicates that settling is also observed in the sediment. Thus, sedimentation of particles in polymer solutions is recursive, since settling is also observed inside the sediment. If all dissipative effects can be neglected, the third concentration wave appears from the very beginning; see Figure 3.

**Figure 4:**
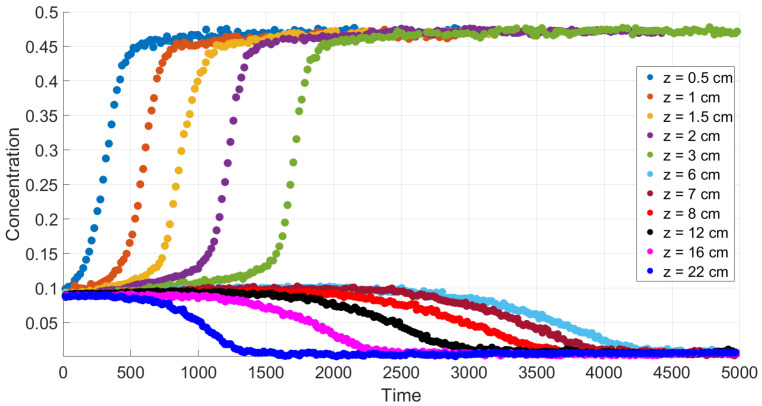
Experimental data for mean concentration versus time at different heights in the case of a Newtonian fluid [4].

**Figure 5:**
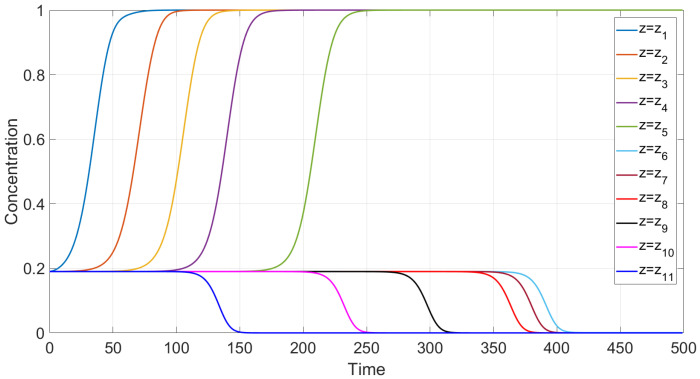
Calculated values of mean concentration versus time at different vertical locations for the flux $F_{rz}$, with m=1.

A comparison with experiment data [4] is performed in Section 6 in the case of small diffusion coefficients. Figure 4 and Figure 5 confirm qualitative agreement when we simulate settling in the Newtonian fluid starting from Equations (24)–(27) with n=1, using the mobility $B_{rz}\left(c\right)$. The main feature of this case is monotonicity in the following sense. Both the experiment data and our calculations reveal that there is a critical height such that at each level above this height, the concentration decreases with time, and at each level below this height, the concentration increases with time.

**Figure 6:**
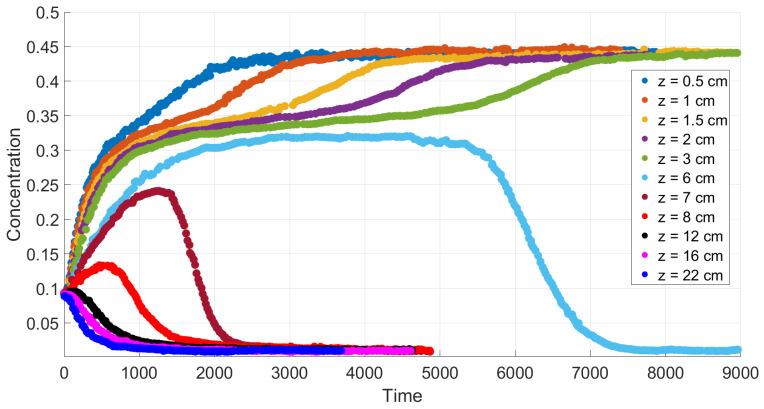
Experimental values of average concentration versus time at different height levels in a polymer solution [4].

**Figure 7:**
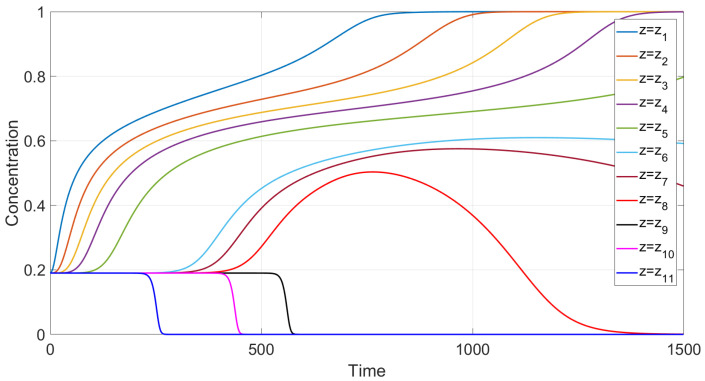
Calculated values of the average concentration versus time at different vertical locations for the shear thinning flux $F_{sh}\left(c\right)$.

Simulation of settling in non-Newtonian shear thinning fluids is based on Equations (24)–(27), with n=0.34 and $B\left(c\right)=B_{sh}\left(c\right)$. Qualitative agreement is depicted in Figure 6 and Figure 7. In such a case, the experimental data and our calculations show that the monotonicity property is preserved only for sufficiently high or sufficiently low vertical locations. There are intermediate locations in which concentration first increases with time and then decreases.

**Figure 8:**
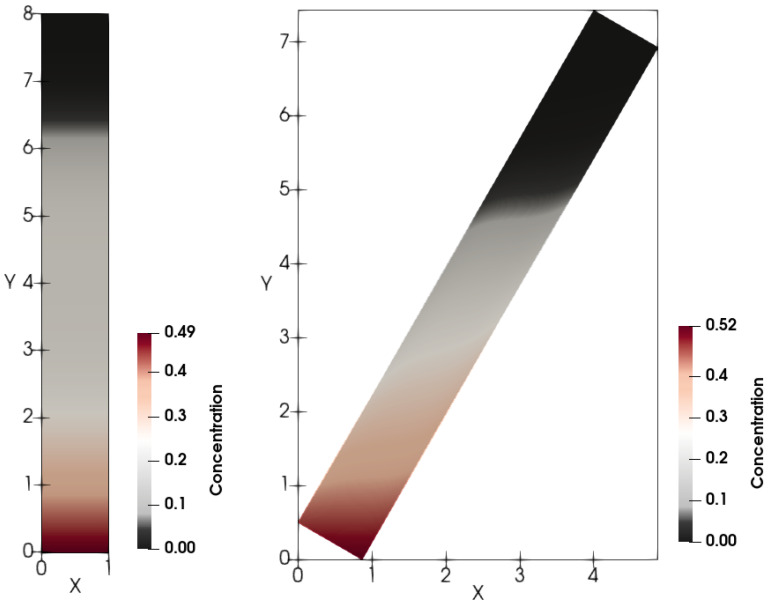
Calculated snapshots of concentration in the case of n=1 for the flux $B_{rz}$ for both the vertical and tilted vessels. The inclination angle is 30°.

**Figure 9:**
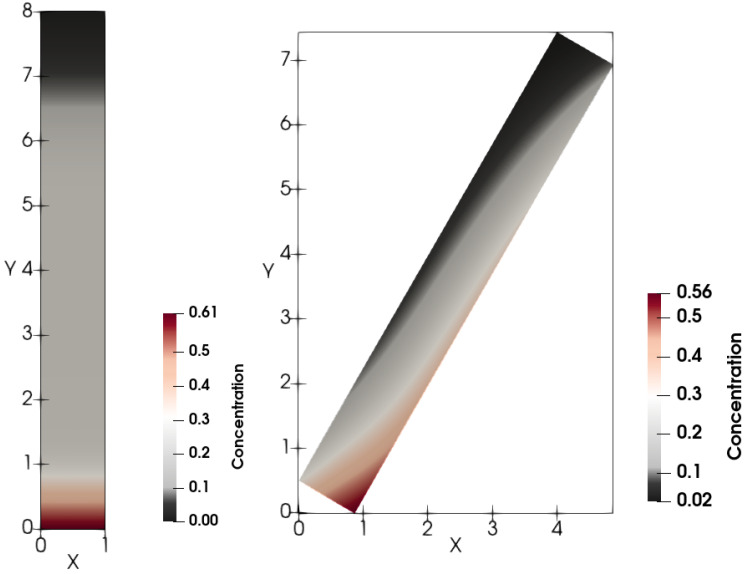
Calculated snapshots of concentration in the case of n=0.34 for the flux $B_{sh}$ for both the vertical and tilted vessels. The inclination angle is 30°.

For both the fluxes $B_{rz}$ and $B_{sh}$, calculations reveal that in spite of no-slip boundary conditions for velocities, horizontal-strata formation in the vertical vessel occurs in the sediment; see Figure 8 and Figure 9.

All the calculations in the present paper are carried out with the use of the open-source PDE Solver FreeFEM++ based on the finite element method. To obey the restriction that the mean volume velocity is divergence-free, the method of artificial compressibility is applied. At each time step, the concentration field is calculated by the Galerkin-characteristics method, which is implemented in FreeFEM++ through the “convect” function. Next, the Navier–Stokes equations are solved to define velocity and pressure. To tackle nonlinearity, iterations are carried out until convergence. Then, a transition is made to the next time step. A weak formulation of the problem and a detailed description of the algorithm are given in Ref. [10]. The ParaView open-source package visualization application is used to visualize the results.

In experiments with blood, A. Boycott (1920) noticed that erythrocyte particles settled faster in an inclined test tube than in a vertical one. Since that time, many attempts have been made to explain this effect in terms of the theory of particle motion in a viscous fluid. For this purpose, additional hydrodynamic forces were introduced, such as the Archimedes force, the Magnus force, etc. Forces were even used that obviously depended on time and on prehistory, such as the Basset–Boussinesq force [27]. In our recent work [10], the Boycott effect was explained without the use of additional hydrodynamic forces, but only due to the gravitational component of the concentration flux in Fick’s law. As far as we know, the issue of particle settling in non-Newtonian fluids has not been considered theoretically and experimentally in the case of inclined vessels.

Let us consider the issue of particle deposition in a 2D tilted tube within the model (24)–(27). Note that this model was used in Ref. [10] in a particular case under the rheological assumptions that $B=B_{rz}$ and n=1 in (7). Now, we set $B=B_{sh}$. As in Ref. [4], calculations are performed for the case n=0.34. The data $B_{rz}$ and n=1 correspond to a Newtonian fluid, whereas the data $B_{sh}$ and n=0.34 correspond to a non-Newtonian shear thinning fluid.

Figure 8 and Figure 9 depict calculated snapshots of concentration in the tilted 2D tube with the inclination angle $\alpha =30^{{}^{\circ}}$. The above clear area extends differently in the Newtonian and non-Newtonian cases. First, the settling is faster in the Newtonian fluid than in the non-Newtonian one.

**Figure 10:**
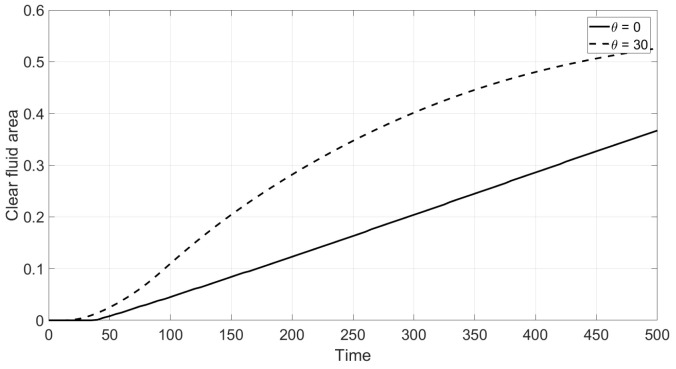
Reduced volumetric rate V(t) of the clear fluid zone c=0 versus dimensionless time for the inclination angles $\alpha =30^{{}^{\circ}}$ from the bottom upwards: Newtonian fluid.

**Figure 11:**
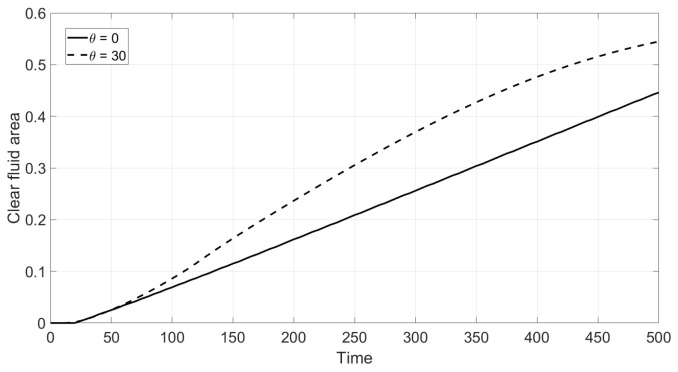
Reduced volumetric rate V(t) of the clear fluid zone c=0 versus dimensionless time for the inclination angles $\alpha =30^{{}^{\circ}}$ from the bottom upwards: non-Newtonian fluid.

Let us introduce the reduced volume of the clear fluid region

(37) $$V\left(t\right)=\frac{1}{a_{1}a_{2}}\int_{\Omega_{\alpha }}1_{c(x,y,t)=0}\;dxdy,$$

where $1_{A}\left(x,y\right)$ is the characteristic function of the set *A*. Figure 10 corresponds to the Newtonian fluid and shows how V(t) depends on time for the vertical and inclined cells. Enhanced sedimentation is observed due to inclination in agreement with the Boycott effect [28]. The result of inclination in the case of the non-Newtonian fluid is shown in Figure 11. This implies that the Boycott effect also takes place in the non-Newtonian shear thinning fluid.

**Figure 12:**
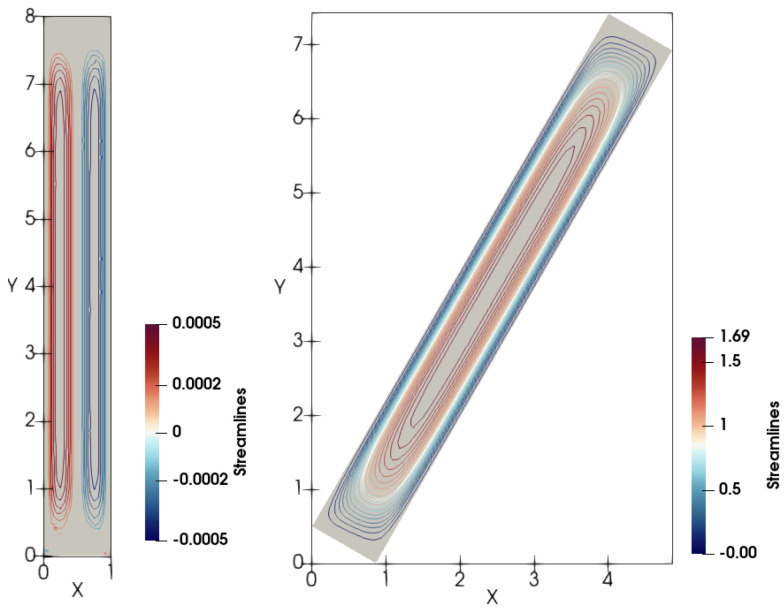
Streamlines of the mean volume velocity v for the vertical and inclined cells in the case of non-Newtonian fluid.

Streamlines of the mean volume velocity v of the non-Newtonian fluid, which define transport of the concentration *c* in the vertical cell, are shown in Figure 12 for some time instant. There are two identical macro-vortices rotating in opposite directions. Due to the lateral particle migration, the lower boundary of the clear fluid zone c = 0 is horizontal at any time. In the case of the tilted cell, one of these vortices becomes dominated and it is in excess of each vertical vortices. Such vortex pattern was observed in experiments by Kinosita [29] and Hill et al. [30].

The recent paper [10] is dedicated to the settling of particles in a Newtonian fluid inside a 2D tube. In this paper, the comparison with experimental data and calculations is given in great detail. One of the results of this work is an explanation of the Boycott effect occurring in the tilted vessel.

## 6. Kinematic Sedimentation Equation

One can process the Moreira [4] experimental data in Figure 6, which show the dependence of particle concentration on time at different heights of a vertical vessel. As a result of the graphical work of converting one data set to another, concentration profiles can be obtained at various time instants. It turns out that the profiles have a smoothed three-wave structure; one wave goes from top to bottom, and the other two from bottom to top. It should be recalled here that there is a classical Kynch sedimentation theory based on a one-dimensional equation for concentration waves [20]. In this approach, only vertical velocities are taken into account, while transverse velocities are considered negligible. The theory has many applications, including sedimentation with absorption [22]. The Kynch equation can be explained in different ways. In fact, it can be derived from the 3D-system (24)–(27). To do this, it suffices to neglect dissipative effects.

Indeed, let us address vertical flows along the variable *y*, 0<y<h, in the case of neutrally buoyant particles. It results from the assumption ${\bar{\rho }}_{s}={\bar{\rho }}_{f}$ that the mean volume velocity and the mean mass velocities coincide. Because of the equation $\mathrm{div}\;\tilde{v}=0$, the vertical component $\tilde{v}$ of $\tilde{v}$ satisfies the equation $\partial \tilde{v}/\partial y=0$. At the same time, $\tilde{v}=0$ at the bottom y=0. Hence, $\tilde{v}=0$ and Equation (31) reduces to the Kynch equation

(38) $$\frac{\partial c}{\partial t}+\frac{\partial F(c)}{\partial y}=0,\;\mathrm{with}\;F=-gcB\left(c\right),$$

where $gB=-v_{slip}$ and $v_{slip}=-V_{St}H\left(c\right)$. The solutions of the Kynch equation (38) are determined mainly by the empirical gravitational mobility B(c). A review of the literature shows that the theoretical three-wave structure can be obtained with a special choice of B(c), [31,32,33].

To describe such a choice, we first consider settling in a Newtonian fluid. Normally, the slip velocity should be determined experimentally. The Richardson–Zaki correlation

(39) $$v_{slip}=-V_{St}H_{rz}\left(c\right),\;H_{rz}\left(c\right)={\left(1-c\right)}^{m},$$

meets experiments with m=4.65 in the case of Newtonian fluid flows with small terminal Reynolds numbers $Re_{t}<<1$ [26].

Let us set $V_{St}=1$ for simplicity. This condition is equivalent to switching to another time scale. Given a vanishing diffusion ε, the settling problem reduces to the following initial boundary value problem:

(40) $$\frac{\partial c}{\partial t}+\frac{\partial F(c)}{\partial y}=\varepsilon \frac{\partial^{2}c}{\partial y^{2}},\;F=-cH\left(c\right),\;0<y<1,$$

with initial and boundary conditions

(41) $${c|}_{t=0}=c_{0}{,\;l|}_{y=0,y=1}=0,$$

where $l=\varepsilon c_{y}-cv_{slip}$. For a more general class of functions F(c) than $F_{rz}\left(c\right)$, we refer the reader to [21] for the study of Equation (40).

**Figure 13:**
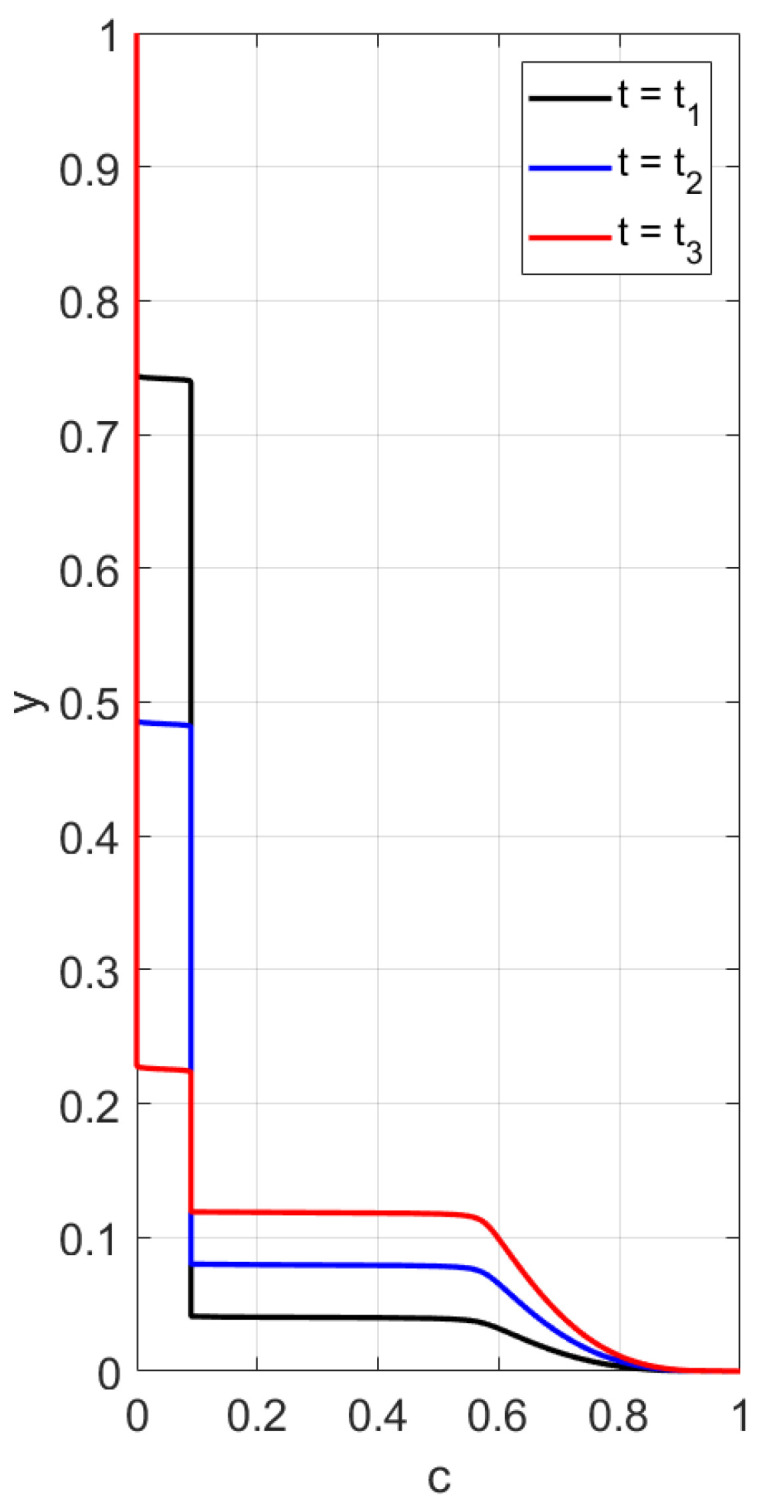
Calculated profiles of the concentration wave at different time instances for the flux $F_{rz}\left(c\right)$ with m=4.65. The initial and boundary data are $c_{0}=0.09$, $c_{bot}=1$, $c_{top}=0$. The rising discontinuity wave is followed by a centered rarefaction wave.

**Figure 14:**
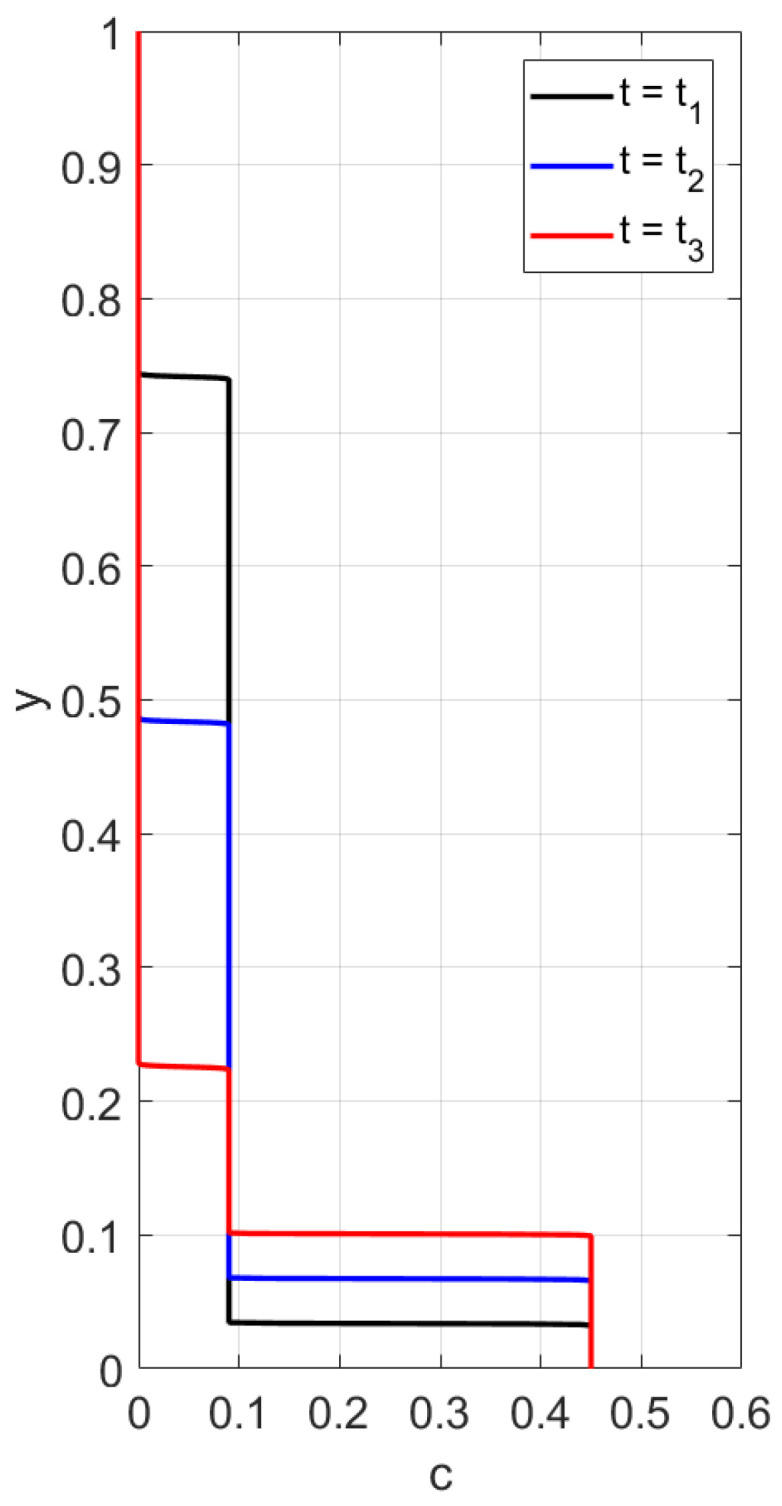
Calculated profiles of the concentration wave at different time instances for the flux $F_{rz}\left(c\right)$ with m=4.65. The initial and boundary data are $c_{0}=0.09$, $c_{bot}=0.45$, $c_{top}=0$. The rising discontinuity wave is not followed by a centered rarefaction wave.

First, we consider the flux $F_{rz}\left(c\right)$ and perform calculations of the concentration profile versus the vertical variable *y*, 0<y<1, at different time instances. The boundary conditions

(42) $${c|}_{y=0}=c_{bot}{,\;c|}_{y=1}=c_{top}$$

are alternative to (41). The results in Figure 13 and Figure 14 are obtained for the conditions (42). One can observe two discontinuity waves propagating towards each other. There are no particles left behind a wave moving from above. The upward wave describes an increase in the height of the sediment. The data for Figure 13 and Figure 14 differ only in the bottom value of concentration. However, the corresponding concentration waves have different structures. When $c_{bot}=0.45$, the rising shock-wave starting from the bottom is followed by a center rarefaction wave; see Figure 13. This is not the case when $c_{bot}=1$; see Figure 14. Concluding the discussion of sedimentation with the function $F_{rz}\left(c\right)$, we note that experiments on particles settling in Newtonian fluids sometimes lead to a three-wave structure [31]. This implies that the flux $F_{rz}\left(c\right)$ is not a unique choice for settling in Newtonian fluids.

Figure 4 and Figure 5 show good qualitative agreement of our calculations with available experiment data for Newtonian fluids [4], provided we use the flux $F_{rz}\left(c\right)$ in the boundary value problem (40), (41). Each curve in these figures corresponds to a time variation of concentration at a fixed value of the vertical variable. Although a quantitative comparison with these experiments is not possible due to the 1D assumption of the present simulations, the results of calculations generally reproduce the experimental trends.

According to the experiment data in the case of a Newtonian fluid (Figure 4), sedimentation in the 30.1 cm-long tube proceeds monotonously in the following sense. The mean concentration at the level *y* decreases in time at each point *y* above the height y≃ 6 cm and it increases in time at each point *y* below the height y≃ 6 cm. The settling loses this monotonicity property as far as the polymer solutions are concerned; see Figure 6. Indeed, one can see that there is an intermediate vertical layer $h_{1}<y<h_{2}$ such that the concentration c(y,t) first increases and then decreases in time for the height *y* from this layer.

To address such issues of monotonicity in the case of shear thinning fluids, we argue as in Ref. [21] and look for the settling velocity correlation in the form

(43) $$v_{slip}\left(c\right)=-V_{St}H_{sh}\left(c\right),$$

(44) $$F_{sh}\left(c\right)=-V_{St}cH_{sh}\left(c\right).$$

with

(45) $$H_{sh}\left(c\right)=a{\left(1-c\right)}^{k}+bc^{m-1}\left(1-c\right)-d{\left(1-c\right)}^{l}.$$

It is assumed for simplicity that $V_{St}=1$. Applying the least squares method for minimization of a discrepancy functional, we find that the values

(46) a=b=1,d=0.95,n=4,m=10,l=8

meet the data in Figure 6 related to the shear thinning fluids [4]. The discrepancy functional determines how close the data are in Figure 6 and Figure 7. Figure 7 is based on solving the initial boundary value problem (40), (41) with $F=F_{sh}$. Remember that the restriction $V_{St}=1$ is equivalent to switching to another time scale. Clearly, the data (46) depend on the time scale.

We performed calculations of concentration profile versus the vertical variable *y*, 0<y<1, at different time instances; see Figure 3. Contrary to the case with the flux $F_{rz}\left(c\right)$, one can observe three discontinuity waves. One wave goes top-down, and the other two rise up, following one after the other. This implies that one more sedimentation occurs within the sediment.

**Figure 15:**
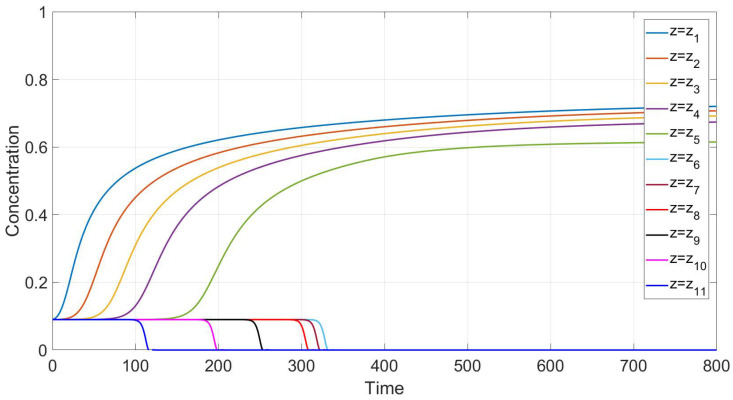
Calculated values of concentration versus time at different vertical locations for the flux $F_{rz}$. There is monotonicity with the initial and boundary data $c_{0}=0.09$, $c_{bot}=1$, $c_{top}=0$.

**Figure 16:**
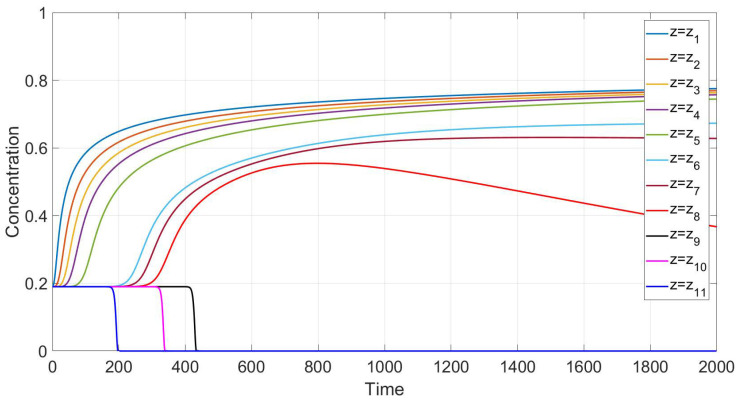
Calculated values of concentration versus time at different vertical locations for the flux $F_{rz}$. Loss of monotonicity with the initial and boundary data $c_{0}=0.19$, $c_{bot}=1$, $c_{top}=0$.

It is worth to remark that loss of monotonicity is also observed for the flux $F_{rz}$. Figure 15 and Figure 16 show that, for some initial data, there is monotonicity, but for other initial data, this property disappears. However, the flux $F_{sh}$ is preferred for simulation of settling in non-Newtonian fluids, as it is consistent with the effect of two-fold particle sedimentation.

The physical meaning of violation of the monotonicity property is as follows. There is a middle layer of the vessel where, at first, the concentration increases due to the sediment going from bottom to top. Then, the concentration in this layer decreases due to a sufficiently pure liquid going from top to bottom. Thus, this effect is the result of the interaction of waves traveling in opposite directions.

Let us summarize the arguments formulated in favor of the new choice (45) for the gravitational mobility $B\left(c\right)=V_{St}H_{sh}\left(c\right)/g$. First of all, our method has a physical background. For a wide variety of coefficients, the equation (45) guarantees the experimentally observed three-wave structure of the concentration profile. From top to bottom, there is a discontinuity wave, followed by a rarefaction wave. From the bottom-up, there are two waves of discontinuity. In addition, the choice of (45) is consistent with the violation of the monotonicity property, which is also observed in experiments. An optimal quantitative choice of coefficients in Equation (45) is achieved by minimizing the discrepancy functional. By comparing the data in Figure 6 and Figure 7, this functional determines how experimental and calculated data are close.

## 7. Conclusions

A two-continua mathematical model for description of particles settling in a shear thinning polymer solution is developed. A correlation formula for the gravitational mobility in Fick’s law is derived and numerical results on sedimentation are consistent with experiments on settling of particles in polymer solutions. In fact, we justify a suitable correlation for the dependence of mobility on particle concentration. The main arguments are based on the Kynch (1952) theory, in which sedimentation of dense suspension is considered as a concentration wave. A zone with a high concentration of particles is first formed at the bottom of a vessel; then, this zone propagates upward like a shock wave. From our calculations, sedimentation of particles in polymer solutions is recursive, since settling is also observed inside the sediment. Sedimentation in a tilted vessel is addressed and it is proven that the Boycott effect also holds in a polymer solution. Comparison with Newtonian carrier fluid is performed.

## Data Availability

Not applicable.

## Abbreviations

*V*	arbitrary volume of suspension, $\left[m^{3}\right]$
$V_{f}$	volume of fluid part in arbitrary volume *V*, $\left[m^{3}\right]$
$V_{p}$	volume of dry particles in arbitrary volume *V*, $\left[m^{3}\right]$
$V_{M}$	volume of mud in arbitrary volume *V*, $\left[m^{3}\right]$
$V_{s}$	$=V_{p}+V_{M}$ volume of solid phase in arbitrary volume *V*, $\left[m^{3}\right]$
$m_{f}$	mass of fluid in arbitrary volume *V*, [kg]
$m_{p}$	mass of dry particles in arbitrary volume *V*, [kg]
$m_{M}$	mass of mud in arbitrary volume *V*, [kg]
$m_{s}$	$=m_{p}+m_{M}$ mass of solid phase in arbitrary volume *V*, [kg]
*m*	$=m_{f}+m_{p}+m_{M}$ mass of arbitrary suspension volume *V*,[kg]
*c*	$=\frac{m_{p}}{m}$ mass concentration of particles, dimensionless
${\bar{\rho }}_{f}$	fluid density, $\left[\frac{kg}{m^{3}}\right]$
${\bar{\rho }}_{p}$	density of dry particles, $\left[\frac{kg}{m^{3}}\right]$
${\bar{\rho }}_{M}$	mud density, $\left[\frac{kg}{m^{3}}\right]$
$\phi_{f}$	volume fraction of fluid, dimensionless
$\phi_{p}$	volume fraction of dry particles, dimensionless
$\phi_{M}$	volume fraction of mud, dimensionless
$\phi_{s}$	$=\phi_{p}+\phi_{M}$ volume fraction of solid phase, dimensionless
$\rho_{f}$	=$\phi_{f}{\bar{\rho }}_{f}$ partial fluid density, $\left[\frac{kg}{m^{3}}\right]$
$\rho_{p}$	$=\phi_{p}{\bar{\rho }}_{p}$ partial particle density, $\left[\frac{kg}{m^{3}}\right]$
$\rho_{M}$	$=\phi_{M}{\bar{\rho }}_{M}$ partial mud density, $\left[\frac{kg}{m^{3}}\right]$
$\rho_{s}$	$=\rho_{p}+\rho_{M}$ partial density of solid phase, $\left[\frac{kg}{m^{3}}\right]$
ρ	$=\rho_{f}+\rho_{p}+\rho_{M}$ total density $\left[\frac{kg}{m^{3}}\right]$
${\bar{\rho }}_{s}$	$=\frac{\rho_{s}}{\phi_{s}}$, density of the solid phase, $\left[\frac{kg}{m^{3}}\right]$
$R_{0}$	$=\frac{{\bar{\rho }}_{s}}{{\bar{\rho }}_{f}}$, ratio of densities, dimensionless
*g*	gravitational acceleration, $980\left[\frac{cm}{s^{2}}\right]$
*p*	pressure, [Pa]
$v_{f}$	velocity of fluid phase, $\left[\frac{m}{s}\right]$
$v_{s}$	velocity of solid phase, $\left[\frac{m}{s}\right]$
u	$=v_{s}-v_{f}$ difference of velocities, $\left[\frac{m}{s}\right]$
v	$=\phi_{f}v_{f}+\phi_{s}v_{s}$ mean volume velocity, $\left[\frac{m}{s}\right]$
$\tilde{v}$	$=\left(1-c\right)v_{f}+cv_{s}$ mean mass velocity, $\left[\frac{m}{s}\right]$
$T_{f}$	viscous part of stress tensor of fluid phase, [Pa]
$T_{s}$	viscous part of stress tensor of solid phase, [Pa]
*D*	rate of strain tensor, $\left[s^{-1}\right]$
*I*	identity matrix, dimensionless
j	total momentum, $\left[\frac{kg}{m^{2}\cdot s}\right]$
l	flux of mass concentration of particles, $\left[\frac{kg}{m^{2}\cdot s}\right]$
$\eta_{f}$	dynamic viscosity of fluid phase, [cp]
$\eta_{s}$	dynamic viscosity of solid phase, [cp]
$\eta_{s}^{0}$	consistency of solid phase, [cp]
τ	shear stress, [cp/s]
$\dot{\gamma }$	dimensionless shear strain
*k*	interphase friction, $\left[\frac{kg}{m^{3}\cdot s}\right]$
*B*	gravitation mobility, [s]
$V_{St}$	Stokes settling velocity, $\left[\frac{m}{s}\right]$
H(c)	hindered settling function, dimensionless
$\frac{d_{s}}{dt}$	differential operator of material derivative related to velocity $v_{s}$
$\frac{d_{f}}{dt}$	differential operator of material derivative related to velocity $v_{f}$
$\frac{d}{dt}$	differential operator of material derivative related to mean mass velocity $\tilde{v}$
$r_{f}$	$=\rho_{f}/{\bar{\rho }}_{f}$, dimensionless density of fluid phase
$r_{s}$	$=\rho_{s}/{\bar{\rho }}_{f}$, dimensionless density of solid phase
*l*	dimensionless length of channel
Re	Reynolds number, dimensionless
Fr	Froude number, dimensionless

## References

[1] Barnes H.A. (2003). Review of the rheology of filled viscoelastic systems. Rheol. Rev..

[2] Chhabra R.P. (2007). Bubbles, Drops, and Particles in Non-Newtonian Fluids.

[3] D’Avino G., Greco F., Maffettone P.L. (2017). Particle migration due to viscoelasticity of the suspending liquid and its relevance in microfluidic devices. Annu. Rev. Fluid Mech..

[4] Moreira B.A., Arouca F.O., Damasceno J.J.R. (2017). Analysis of suspension sedimentation in fluids with rheological shear-thinning properties and thixotropic effects. Powder Technol..

[5] Datt C., Elfring G.J. (2018). Dynamics and rheology of particles in shear-thinning fluids. J.-Non-Newton. Fluid Mech..

[6] Buscall R., Goowin J.W., Ottewill R.H. (1982). The settling of particles through Newtonian and non-Newtonian media. J. Colloid Interface Sci..

[7] Oblak B., Babnik S., Erklavec-Zajec V., Likozar B., Pohar A. (2020). Digital twinning process for stirred tank reactors/Separation unit operations through tandem experimental/Computational Fluid Dynamics (CFD) Simulations. Processes.

[8] Pohar A., Likozar B. (2014). Dissolution, Nucleation, Crystal Growth, Crystal Aggregation, and Particle Breakage of Amlodipine Salts: Modeling Crystallization Kinetics and Thermodynamic Equilibrium, Scale-up, and Optimization. Ind. Eng. Chem. Res..

[9] Pohar A., Naneh O., Bajec D., Likozar B. (2020). Chemical reactor/compounding vessel fingerprinting: Scale-up/down considerations for homogeneous and heterogeneous mixing using computational fluid dynamics. Chem. Eng. Res. Des..

[10] Shelukhin V.V., Neverov V.V. (2022). Dense suspension flows: A mathematical model consistent with thermodynamics. J. Fluids Eng. ASME.

[11] Morris J.F., Boulay F. (1999). Microstructure of strongly sheared suspensions and its impact on rheology and diffusion. J. Rheol..

[12] Baumgarten A.S., Kamrin K. (2019). A general fluid-sediment mixture model and constitutive theory validated in many flow regimes. J. Fluid Mech..

[13] Khalatnikov I.M. (1989). An Introduction to the Theory of Superfluidity.

[14] Landau L.D., Lifshits E.M. (1987). Fluid mechabics. Course of Theoretical Physics.

[15] DeGroot S.R., Mazur P. (1962). Non Equilibrium Thermodynamics.

[16] Blokhin A.M., Dorovskii V.N. (1995). Mathematical Modelling in the Theory of Multivelocity Continuum.

[17] Dorovskii V.N., Perepechko Y.V. (1996). The hydrodynamic model of solution in cracking-porous media. Russ. Geol. Geophys..

[18] Shelukhin V.V. (2014). A poroelastic medium saturated by a two-phase capillary fluid. Contin. Mech. Thermodyn..

[19] Shelukhin V.V. (2018). Thermodynamics of two-phase granular fluids. J.-Non-Newton. Fluid Mech..

[20] Kynch G.F. (1952). A theory of sedimentation. Trans. Faraday Soc..

[21] Bustos M.C., Concha F., Bürger R., Tory E.M. (1999). Sedimentation and Thickening Phenomenological Foundation and Mathematical Theory.

[22] Shelukhin V.V. (2005). Quasistationary sedimentation with adsorption. J. Appl. Mech. Tech. Phys..

[23] Shelukhin V.V. (2021). Rotational particle separation in solutions: Micropolar fluid theory approach. Polymers.

[24] Ishii M., Mishima K. (1984). Two-fluid model and hydrodynamic constitutive relations. Nucl. Eng. Des..

[25] Acrivos A., Herbolzheimer E. (1979). Enhanced sedimentation in settling tanks with inclined walls. J. Fluid Mech..

[26] Richardson J.F., Zaki W.N. (1954). The sedimentation of a suspension of uniform spheres under conditions of viscous flow. Chem. Eng. Sci..

[27] Nevskii Y., Osiptsov A. (2011). Slow gravitational convection of disperse systems in domains with inclined boundaries. Fluid Dyn..

[28] Boycott A.E. (1920). Sedimentation of blood corpuscles. Nature.

[29] Kinosita K. (1949). Sedimentation in tilted vessels. J. Colloid Interface Sci..

[30] Hill W.D., Tothfus R.R., Li K. (1977). Boundary-enhanced sedimentation due to settling convection. Int. J. Multiph. Flow.

[31] Barton N.G., Li C.-H., Spencer S.J. (1992). Control of a surface of discontinuity in continuous thickness. J. Austral. Math. Soc. Ser. B.

[32] Been K., Sills G.C. (1981). Self-weight consolidation of soft soils: An experimental and theoretical study. Geotechnique.

[33] Auzerais F.M., Jackson R., Russel W.B. (1988). The resolution of shocks and the effects of compressible sediments in transient settling. J. Fluid Mech..

